# A bibliometric analysis of 100 top-cited journal articles related to acupuncture regulation of the autonomic nervous system

**DOI:** 10.3389/fnins.2022.1086087

**Published:** 2022-12-22

**Authors:** Zhanhao Zhao, Li Li, Chen Xin, Yaqun Yin, Rong Zhang, Jing Guo

**Affiliations:** ^1^Danyang Hospital of Traditional Chinese Medicine, Teaching Hospital of Nanjing University of Chinese Medicine, Danyang, China; ^2^Acupuncture and Massage College, Health and Rehabilitation College, Nanjing University of Chinese Medicine, Nanjing, China; ^3^Department of Acupuncture Rehabilitation, The Affiliated Hospital of Nanjing University of Chinese Medicine, Nanjing, China; ^4^Institute of Acupuncture and Moxibustion, China Academy of Chinese Medical Sciences, Beijing, China; ^5^School of Health Sciences, Wuhan University, Wuhan, China

**Keywords:** acupuncture, autonomic nervous system, somato-autonomic reflex, bibliometric analysis, citation analysis

## Abstract

**Purpose:**

Research on the effects of acupuncture on autonomic function has been conducted for several decades, and a few notable studies have emerged in recent years. This study used bibliometric analysis to assess 100 top-cited articles to characterize the current status and research trends over the last three decades.

**Methods:**

The 100 top-cited publications were identified from the Web of Science Core Collection database. The bibliometrix package in R was used for quantitative and qualitative analyses of the publication patterns and the country/region, institution, and author contributions. VOSviewer was used to construct networks based on co-citation analysis of the journals and the keyword co-occurrence.

**Results:**

The 100 top-cited articles were identified with a total of 8,123 citations (range: 37–345). The majority of the articles came from the USA (*n* = 42), followed by Japan (*n* = 14) and mainland China (*n* = 13). Articles from the USA exhibited the largest number of citations (3,582 citations), followed by articles from Japan (1,189 citations), then articles from mainland China (755 citations). Neurosciences/Neurology was the most studied research area (*n* = 41). The *Autonomic Neuroscience: Basic and Clinical* published the largest number of papers (*n* = 14), while *Brain Research* received the largest number of citations (205 citations). Longhurst JC was the most productive author (10 publications), and Sato A was first among the cited authors (87 citations). The most frequently cited articles that focused on gastrointestinal, cardiovascular, or gynecologic responses to acupuncture regulation of the autonomic nervous system first appeared in the 1990s, peaked in the 2000s, then decreased after 2010. Publication of articles focused on the anti-inflammatory effects of acupuncture associated with autonomic function demonstrated an increasing trend over the last three decades.

**Conclusion:**

From the initial studies focusing on the autonomic mechanism of visceral responses to acupuncture, researchers concentrated on exploring the autonomic mechanism of acupuncture in the control of systemic inflammation. Non-invasive electrical methods that activate somato-autonomic reflexes are current translational directions in clinical practice. Additional investigation of the underlying neuroanatomical basis of somato-autonomic reflexes also is needed.

## Introduction

Acupuncture, one component of traditional Chinese medicine, has been practiced for thousands of years in China. According to the World Health Organization (WHO) 2019 global report, acupuncture is one of the most commonly used traditional and complementary therapies worldwide (World Health Organization, [WHO], 2019). Based on traditional Chinese medicine concepts, acupuncture aims to rebalance Yin and Yang. It is believed that balanced Yin and Yang maintains body homeostasis by regulating the autonomic nervous system (ANS), which consists of the parasympathetic and sympathetic subdivisions (Takahashi, 2011). It has been documented that acupuncture stimulates somatic afferents to regulate various autonomic functions. Acupuncture activates the somatosensory-autonomic pathways to modulate blood pressure, gastrointestinal motility, and systemic inflammation (Longhurst and Tjen, 2013; Ma, 2020, 2022; Ulloa, 2021; Tjen-A-Looi et al., 2022).

Bibliometric analysis is a broadly used quantitative and qualitative method to analyze published academic literature and can be used to track the development of a specific research field. The number of citations a publication receives is often utilized as a rough measure of its importance. Highly cited papers are considered milestone studies in their specific fields and directly influence the understanding of the field (Van Noorden et al., 2014). Notably, research into the effects of acupuncture on autonomic function has been conducted for several decades, yet few critical studies have emerged in recent years. Therefore, it is necessary to explore the influential articles that have been published and predict future trends. In this study, we conducted bibliometric analyses of the 100 top-cited papers in acupuncture regulation of the ANS over the past three decades to understand past and current research trends. In turn, these results provided a general understanding of clinical and mechanism advances and how acupuncture regulates autonomic function.

## Materials and methods

### Data sources and search strategy

The Web of Science (WoS) Core Collection database, including SCI-EXPANDED was systematically searched on October 10, 2022. The search terms were obtained from the MeSH database via PubMed and relevant references. The search strategy and results are shown in Table 1. There are no restrictions on language and date of publication, while the publication timespan was compiled using Wos SCI-EXPANDED accessed from Wuhan University (1979 to the present).

**TABLE 1:** Search strategy.

Set	Result	Search query
#1	30,536	TS = (*acupuncture* OR acupoint* OR acupressure OR auricular OR acupotom* OR acustimulation)
#2	2,92,094	TS = (autonomic* OR *sympathetic OR vagus OR vagal OR “vegetative nervous system” OR “visceral nervous system” OR acetylcholine OR “cholinergic nerve” OR “adrenergic nerve” OR *adrenaline)
#3	1,499	#1 AND #2
#4	1,361	#1 AND #2 AND Article or Review (document type)

### Data collection

Only original articles and reviews focused on acupuncture’s regulation of the ANS were included in this study. Literature concerning transcutaneous auricular vagus nerve electrical stimulation without auricular points or acupuncture theory was excluded. Two authors (ZZ and RZ) independently searched and screened the 100 most frequently cited articles. Any disagreement was resolved by consensus involving the third author (JG). The selection of the documents gathered for this study is described in Supplementary Figure 1. The final 100 records were downloaded from the WoS as.txt files with “full records and cited references” from the Wos. The following information was extracted from the selected articles: author names, country or regions, article title, publishing journal, year of publication, subject area, keywords, institutions, and the total number of citations. Different topics, annual publications of countries or regions, and annual publications of the seven most productive institutions were independently extracted and summarized by the two authors (ZZ and LL). If any disagreements remained unsolved, the differences were mediated by the third author (JG).

### Bibliometric analysis and visualization

The bibliographic information of the selected publications was converted into Microsoft Excel 2019 (Microsoft Corp., Redmond, WA, USA). The annual publication pattern of countries/regions and institutions, and trends in the top-cited publications for different main topics over time were assessed using Microsoft Excel 2019. Number of total citations per year was also calculated. The journal impact factors were collected from the 2021 Journal Citation Reports (JCR) (Clarivate Analytics, Philadelphia, PA, USA).

The data were analyzed and automatically visualized using the bibliometrix package (version 3.0.3) in R software (version 4.0.3) (Aria and Cuccurullo, 2017). The data included publication patterns by year, research areas, author contributions, and authors’ publication records over time. Lotka’s law also was included, which connotes a prediction that the number of authors contributing a specific number of papers is inversely proportional to the number of papers contributed (Lotka, 1926). We determined whether the author productivity distribution followed the Lotka’s law. The function “lotka” returns a list of summary statistics of the Lotka’s law estimation of an object of the class “bibliometrix” in R. *R*^2^ indicates the goodness of fit. The *P* value of the two-sample Kolmogorov-Smirnov test between the observed distribution and the theoretical Lotka’s law prediction indicated whether the author’s productivity fit Lotka’s law. A *P* ≥ 0.05 indicated that the data fit Lotka’s law.

Co-citation analysis of journals and co-occurrence analysis of keywords were mapped using VOSviewer (Version 1.6.16, Leiden University, the Netherlands) (Van Eck and Waltman, 2010, 2017). Journals that were co-cited in more than 20 publications were mapped. Keywords that occurred more than five times were presented in three visualizations (network, overlay, and density visualization) of the co-occurrence analysis to identify essential terms associated with the effect of acupuncture on autonomic function.

## Results

### Citation count and publication period

**FIGURE 1:**
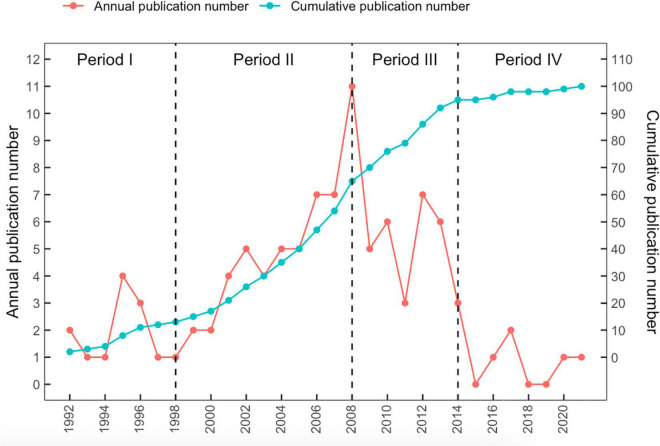
Number of top-cited 100 publications by year.

A total of 1,361 articles on the regulation of ANS by acupuncture were identified in the WoS Core Collection database. The 100 top cited articles are listed in Supplementary Table 1 in descending order based on each article’s total number of citations. The number of citations ranged from 37 to 345. The earliest articles in the list were two articles published in 1992. The two most recent articles were published in *Neuron* (2020) and *Nature* (2021). As noted in Figure 1, publications in the field from 1998 to 2008 exhibited a robust increase in number. The year that yielded the highest number of influential articles was 2008 (*n* = 11). In addition, 10 most influential articles are listed in Table 2 based on the number of total citations per year.

**TABLE 2:** 10 most influential articles based on total citations per year from 100 top-cited articles.

Rank	Article	TC per year
1	Liu S, et al. A neuroanatomical basis for electroacupuncture to drive the vagal-adrenal axis. *Nature*. 2021;598(7882):641–645.	57
2	Zhang R, et al. Mechanisms of acupuncture-electroacupuncture on persistent pain. *Anesthesiology*. 2014;120(2):482–503.	43.13
3	Torres-Rosas R, et al. Dopamine mediates vagal modulation of the immune system by electroacupuncture. *Nat Med*. 2014;20(3):291–5.	39.63
4	Liu S, et al. Somatotopic Organization and Intensity Dependence in Driving Distinct NPY-Expressing Sympathetic Pathways by Electroacupuncture. *Neuron*. 2020;108(3):436–450.e7.	30
5	Dhond RP, et al. Acupuncture modulates resting state connectivity in default and sensorimotor brain networks. *Pain*. 2008;136(3):407–418.	14.93
6	Lin JG, et al. Acupuncture analgesia: a review of its mechanisms of actions. *Am J Chin Med*. 2008;36(4):635–45.	14.57
7	Ulloa L, et al. Nerve Stimulation: Immunomodulation and Control of Inflammation. *Trends Mol Med*. 2017;23(12):1103–1120.	12.6
8	Jin H, et al. Anti-inflammatory effects and mechanisms of vagal nerve stimulation combined with electroacupuncture in a rodent model of TNBS-induced colitis. *Am J Physiol Gastrointest Liver Physiol*. 2017;313(3):G192–G202.	12.6
9	Lim HD, et al. Anti-Inflammatory Effects of Acupuncture Stimulation via the Vagus Nerve. *PLoS One*. 2016;11(3):e0151882.	12.17
10	Andersson S, et al. Acupuncture – from empiricism to science: functional background to acupuncture effects in pain and disease. *Med Hypotheses*. 1995;45(3):271–81.	11.96

TC, total citation.

### Distribution of countries/regions and institutions

**FIGURE 2:**
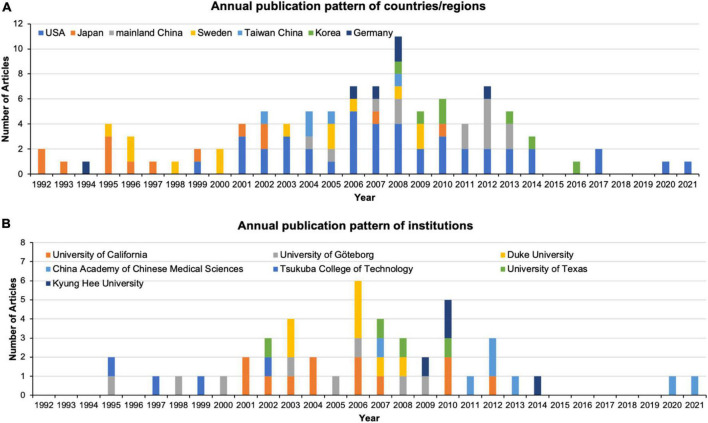
Annual publication pattern of **(A)** countries/regions; **(B)** institutions.

As is noted in Table 3 and Figure 2A, corresponding authors in seven countries/regions of corresponding authors contributed to the 100 most cited publications in this field. The USA contributed the most articles (*n* = 42), followed by Japan (*n* = 14), mainland China (*n* = 13), Sweden (*n* = 13), South Korea (*n* = 7), Germany (*n* = 6), and Taiwan (*n* = 5). Articles from the USA had the highest number of citations (3,582 citations), followed by those from Japan (1,189 citations) and mainland China (755 citations). Sweden has the highest ratio of total citations (105.38), followed by Taiwan (99.4) and the USA (85.29).

**TABLE 3:** Number of publication and total citation by country/region.

Country/Region	Publication	TC	TC/Publication
USA	42	3,582	85.29
Japan	14	1,189	84.93
Mainland China	13	755	58.08
Sweden	13	1,370	105.38
South Korea	7	409	58.43
Germany	6	321	53.5
Taiwan	5	497	99.4

TC, total citation.

As is shown in Figure 2B, seven institutions contributed 46% of the articles to the 100 most cited publications in this field. The University of California (12 records) contributed the most numbers of publications, followed by the University of Göteborg (8 records), Duke University (7 records), and the China Academy of Chinese Medical Sciences (7 records).

### Analysis of journals and research areas

**FIGURE 3:**
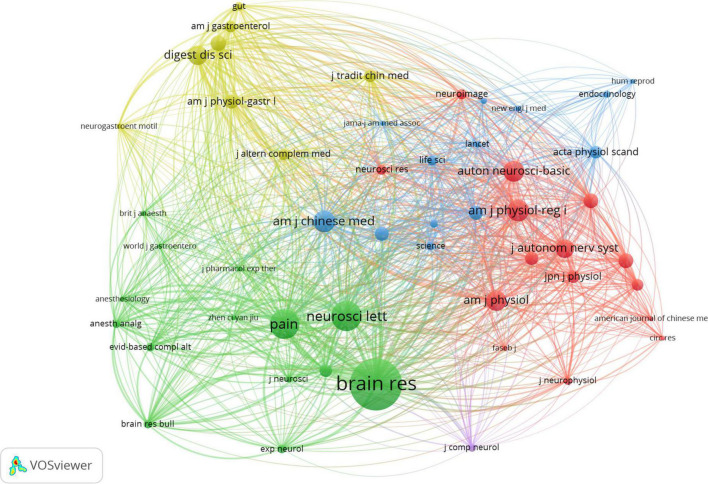
Network map of journals that were co-cited in more than 20 publications.

The 100 top-cited articles were published in 50 journals. Table 4 shows the top 10 most popular journals. The *Autonomic Neuroscience: Basic and Clinical* (14 records) published the most studies, followed by the *American Journal of Physiology-Gastrointestinal and Liver Physiology* (5 records), *American Journal of Physiology-Regulatory, Integrative and Comparative Physiology* (5 records), and *Evidence-Based Complementary and Alternative Medicine* (5 records). As noted in Figure 3, a total of 51 journals co-cited in more than 20 publications were analyzed by VOSviewer. The top 10 cited journals that published related articles are shown in Table 4. *Brain Research* had the largest number of citations (205 citations), followed by *Neuroscience Letters* (119 citations), *Pain* (116 citations), *the American Journal of Chinese Medicine* (84 citations), and the *American Journal of Physiology-Regulatory, Integrative and Comparative Physiology* (84 citations).

**TABLE 4:** Top 10 popular journals and cited journals.

Rank	Popular journals	Records (*n*)	2021 impact factor	2021 JCR partition	Cited journals	Citations (*n*)	2021 impact factor	2021 JCR partition
1	*Auton Neurosci-Basic*	14	2.355	Q4	*Brain Res*	205	3.610	Q3
2	*Am J Physiol-Gastr L*	5	4.871	Q1	*Neurosci Lett*	119	3.197	Q3
3	*Am J Physiol-Reg I*	5	3.210	Q2	*Pain*	116	7.926	Q1
4	*Evid-Based Compl Alt*	5	2.650	Q3	*Am J Chinese Med*	84	6.005	Q1
5	*Am J Chinese Med*	4	6.005	Q1	*Am J Physiol-Reg I*	84	3.210	Q2
6	*Digest Dis Sci*	4	3.487	Q3	*Auton Neurosci-Basic*	83	2.355	Q4
7	*Am J Physiol -Heart C*	3	5.125	Q1	*Am J Physiol*	78	−	–
8	*Anesthesiology*	3	8.986	Q1	*Digest Dis Sci*	76	3.487	Q3
9	*Brain Res Bull*	3	3.715	Q3	*J Autonom Nerv Syst*	71	−	–
10	*J Appl Physiol*	3	3.880	Q2	*Gastroenterology*	60	33.883	Q1

A total of 18 research areas were identified among the 100 top-cited publications. The most highly represented research area was Neurosciences Neurology (41 records), followed by Physiology (19 records), Integrative Complementary Medicine (16 records), Gastroenterology Hepatology (14 records), and Anesthesiology (8 records; Table 5).

**TABLE 5:** Top 10 well-represented research areas.

Rank	Research areas	Records (*n*)	Rank	Research areas	Records (*n*)
1	Neurosciences Neurology	41	6	Research Experimental Medicine	5
2	Physiology	19	7	Cardiovascular System Cardiology	4
3	Integrative Complementary Medicine	16	8	General Internal Medicine	4
4	Gastroenterology Hepatology	14	9	Science Technology Other Topics	4
5	Anesthesiology	8	10	Sport Sciences	4

### Analysis of authors

**FIGURE 4:**
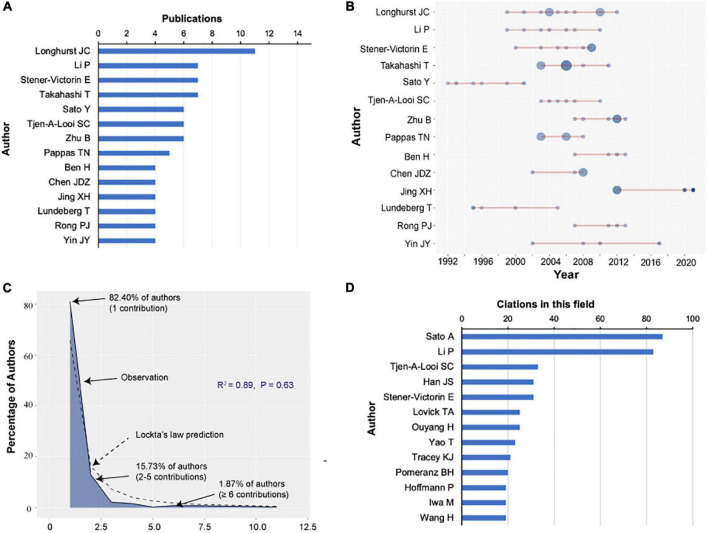
Analysis of authors. **(A)** Number of publications from different authors. **(B)** Total citations in the research filed from different authors. **(C)** Authors’ production over time. **(D)** Lotka’s Law on the contribution of authors who published articles in the field.

Based on the number of publications, Longhurst JC was the most productive author, with 10 articles (10% of all articles), followed by Li P (7%), Stener-Victorin E (7%), Takahashi T (7%), Sato Y (6%), Tjen-A-Looi SC (6%), and Zhu B (6%; Figures 4A,B). Figure 4B also shows the authors’ number of publications over time, with a larger circle representing more publications. 82.40% of authors contributed to one publication, 15.73% participated in 2–5 publications, and 1.87% contributed to 6 or more publications. Therefore, the author productivity in this research area fits Lotka’s law (*R*^2^ = 0.89, *P* = 0.63), which indicated that the number of authors declined as the number of published papers increased (Figure 4C). In analyzing authors’ citations among the 100 top-cited papers in this field, Sato A was ranked first (87 citations), followed by Li P (83 citations), Tjen-A-Looi SC (33 citations), Han JS (31 citations), and Stener-Victorin E (31 citations; Figure 4D).

### Analysis of keywords and topics

**FIGURE 5:**
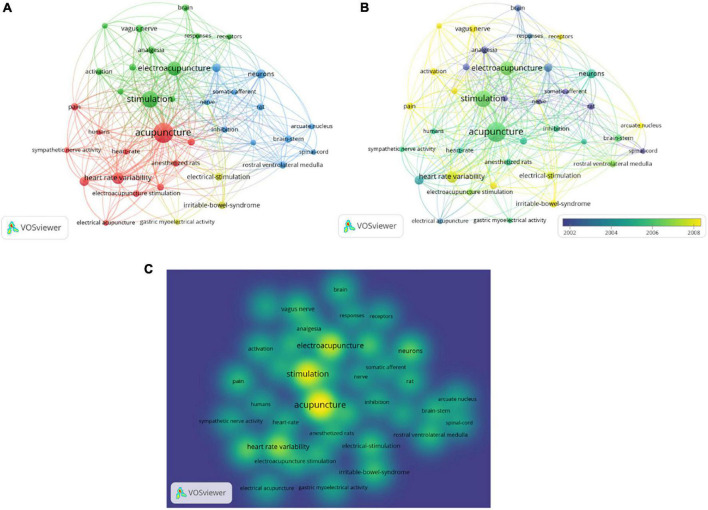
Co-occurrence analysis of keywords. **(A)** Mapping of keywords of studies. **(B)** Distribution of keywords according to average publication year (blue: earlier, yellow: later). **(C)** Distribution of keywords according to the mean frequency of appearance. Keywords in yellow occurred with the highest frequency.

In total, 38 keywords were identified as having co-occurrence more than five times (Figure 5A). As is shown in Figure 5B, the colors in the overlay visualization indicated the average publication year of the identified keywords. Most of the keywords were published after 2002, with greener or yellower colors. The density visualization showed the keywords according to the frequency of appearance (Figure 5C).

**FIGURE 6:**
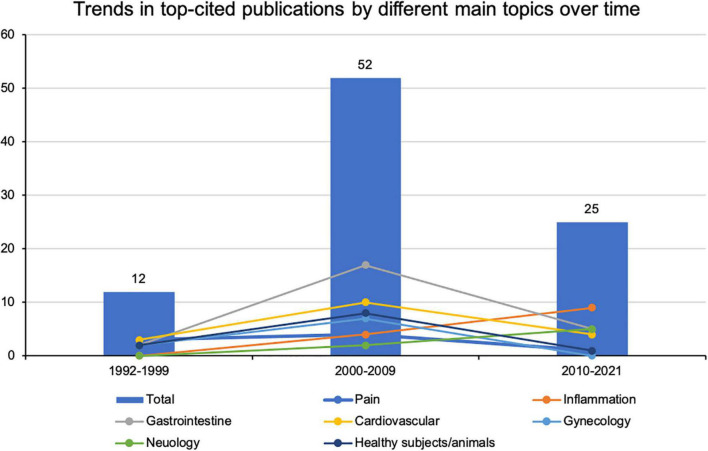
Trends in top-cited publications by different main topics over time.

The most frequently cited articles that focused on gastrointestinal, cardiovascular, or gynecologic responses to acupuncture’s regulation of ANS appeared in the 1990s, peaked in the 2000s and decreased after 2010. Articles focused on the anti-inflammatory effects of acupuncture associated with autonomic function demonstrated an increasing trend over the last three decades (Figure 6).

## Discussion

This study was significant because we identified the top 100 cited articles and their primary characteristics related to acupuncture’s regulation of the ANS. This bibliometric analysis enabled the identification of critical information and provided a historical perspective on scientific progress in the field over the last three decades. The USA had the largest number of publications and citations, followed by Japan, then mainland China. The top-cited article with 345 citations was titled “Mechanisms of acupuncture-electroacupuncture on persistent pain,” authored by Zhang et al. (2014). Although this is a review article focused on the mechanisms of acupuncture’s analgesic effects, it is mentioned that electroacupuncture (EA) activates sympathetic nerve fibers to inhibit inflammation-induced pain and possible biological processes. This activated effect of EA activation on sympathetic nerves might promote the migration of opioid-containing cell migration into inflammatory sites and increase endogenous opioid release. However, the exact mechanism remains unclear and requires additional investigation.

### Cardiovascular responses to acupuncture regulation of autonomic function

Longhurst JC from the University of California had the largest number of publications among the 100 top-cited articles, with 11 articles from 1999 to 2012 (Chao et al., 1999; Li et al., 2001; Tjen-A-Looi et al., 2003; Guo et al., 2004; Tjen-A-Looi et al., 2004; Zhou et al., 2005; Tjen-A-Looi et al., 2006; Tjen-A-Looi et al., 2007; Li and Longhurst, 2010; Moazzami et al., 2010; Zhou and Longhurst, 2012). The other two productive authors (Li P and Tjen-A-Looi SC) are colleagues of Longhurst JC and co-authored several articles concerning the cardiovascular responses to EA’s regulation of autonomic function. They systematically reported the mechanisms of EA’s effects on hypertension and hypotension via autonomic regulation. They indicated that the evoked somatic afferents occurring during acupuncture modify the hyperactive or hypoactive responses in various CNS regions from the hypothalamus paraventricular nucleus (PVN) to brainstem rostral ventrolateral medulla (RVLM) and nucleus ambiguous (NA), and regulate neurotransmitters to improve imbalances in the ANS (Chao et al., 1999; Crisostomo et al., 2005; Li et al., 2010; Moazzami et al., 2010; Longhurst and Tjen, 2013; Tjen-A-Looi et al., 2022).

Low-current and low-frequency EA could reduce elevated blood pressure in mild to moderate hypertension patients with long-lasting effects (Chao et al., 1999; Li et al., 2015). EA can inhibit the activated sympathoexcitatory reflex and increase sympathetic tone resulting in reductions in elevated blood pressure (Tjen-A-Looi et al., 2006; Li et al., 2015). Underlying mechanisms driving the EA hypotensive response are orchestrated by neurotransmitters (glutamate, acetylcholine, opioids, GABA, nociceptin, serotonin and endocannabinoids), which activate their specific receptors to reduce the increased sympathetic outflow and participate in the EA-related CNS long-loop neuronal pathway and circuitry (Tjen-A-Looi et al., 2022). EA stimulation at *Jianshi* (PC5), *Neiguan* (PC6), *Zusanli* (ST36), and *Shangjuxu* (ST37) acupoints significantly reversed the phenylbiguanide-induced bradycardia and hypotension (vasovagal reflex responses), in part through the actions of GABA and opioids in the nucleus tractus solitarius (NTS) (Tjen-A-Looi et al., 2018).

### Gastrointestinal responses to acupuncture regulation of autonomic function

Acupuncture has been reported to modulate imbalances between the parasympathetic and sympathetic activity to regulate gastrointestinal (GI) motility effectively. Sato A was ranked first in authors’ citations among the 100 top-cited papers. Sato A and colleagues focused on investigating somatosensory input in autonomic functions. Their publication titled “Neural mechanisms of the reflex inhibition and excitation of gastric motility elicited by acupuncture-like stimulation in anesthetized rats (1993)” was the most co-cited reference in our analysis (Sato et al., 1993). They demonstrated that acupuncture-like stimulation in the abdominal region elicited gastric inhibitory responses via the abdominal afferent nerve- spinal-sympathetic pathway. The excitatory gastric response was evoked by acupuncture-like stimulation at the hind paw region via the hind paw afferent nerve-brain-vagal pathway (Sato et al., 1993). Zhu B and colleagues reported that acupuncture at acupoints homo-segmental to the region of gastric innervation activates sympathetic nerves resulting in an inhibitory response in gastric motility. Acupuncture at hetero-segmental acupoints induced vagal nerve activity to accelerate gastric motility (Li et al., 2007).

Takahashi T has contributed seven (Tada et al., 2003; Tatewaki et al., 2003; Iwa et al., 2006a,b; Takahashi, 2006, 2011; Imai et al., 2008) publications among the 100 top-cited articles. Takahashi T and colleagues found that EA at ST36 stimulated motility and transit in the distal colon but not the proximal colon, in freely moving conscious rats. The stimulatory effect was abolished by pretreatment with atropine or extrinsic nerve denervation extrinsic nerves associated with the distal colon. The effect of EA at ST36 on accelerated distal colonic motility and transit was associated with activation of the sacral parasympathetic-pelvic pathway and Barrington’s nucleus (Iwa et al., 2006a). Dual effects of EA on GI motility in stressful conditions were observed with stimulation of delayed gastric emptying via cholinergic pathways and inhibition of accelerated colonic transit via the sympathetic pathway (Iwa et al., 2006b). They also reported that the application of EA at different body regions (forelimb/hindlimb acupoints versus abdominal acupoints) activated different autonomic pathways associated with GI-motility control. EA at ST36 accelerates gastric motility via parasympathetic reflexes (somatosensory-NTS-DMV-parasympathetic efferent pathway), while EA at *Tianshu* (ST25) inhibits gastric motility via sympathetic reflexes (somatosensory-NTS-RVLM-sympathetic efferent pathway) (Iwa et al., 2007; Takahashi, 2011).

Chen JDZ from the University of Michigan has published four (Ouyang et al., 2002; Sallam et al., 2007; Chen et al., 2008; Liu et al., 2008) top-cited articles in this field. Like Takahashi T’s lab, Chen JDZ and colleagues focused on the effect of EA on GI motility via parasympathetic and sympathetic pathways. Using a canine gastroparesis model, they found that EA at PC6 and ST36 increased gastric slow-wave rhythmicity in both the proximal and distal stomach and myoelectrical spike activity in the distal stomach that accelerated the gastric emptying of liquid. Enhanced peripheral vagal activity assessed from the spectral analysis of heart rate variability after EA intervention indicated the possible involvement of the vagal pathway in regulating gastric motility by EA (Ouyang et al., 2002). The ameliorating effects of EA on rectal distension-induced gastric slow-wave and antral contractions might have occurred through enhanced vagal activity, which was partially blocked by naloxone (Chen et al., 2008). They applied transcutaneous electroacupuncture/electrical acustimulation (TEA) in clinical practice, which is a non-invasive method that stimulates acupoints via surface electrodes instead of needles. TEA application at PC6 and ST36 decreased GI symptom scores and restored the sympathetic-vagal balance in systemic sclerosis patients and functional dyspepsia patients (Sallam et al., 2007; Liu et al., 2008). Two recent studies reported that TEA increased vagal activity in patients with irritable bowel syndrome with constipation or gastroesophageal reflux disease, and the increase correlated with several GI symptoms (Zhang et al., 2021; Huang et al., 2022).

### Acupuncture activates somato-autonomic reflexes to modulate systemic inflammation

EA also has been demonstrated to activate somato-autonomic pathways that could modulate systemic inflammation or cytokine storms. Nine reviews or articles of animal studies included in the 100 top-cited articles demonstrated that acupuncture could control systemic inflammation via ANS regulation. In 2004, Tracey KJ emphasized the cholinergic anti-inflammatory pathway (the inflammatory reflex) (Tracey, 2002) and reported that inflammatory mediators activate afferent signals, which are relayed to the NTS. Subsequent activation of vagus efferent activity leads to acetylcholine (ACh) release that binds to α7 nicotinic acetylcholine receptors (α7nAChR) on tissue macrophages, which inhibit the activation of macrophages and cytokine release. Acupuncture is a promising approach to modulate vagus nerve activity in the control of systemic inflammation. Still, the relationships between the stimulatory effects of acupuncture on vagus nerve activity and anti-inflammatory action have not been clearly defined previously (Tracey, 2002).

Ulloa L and colleagues published “Dopamine mediates vagal modulation of the immune system by electroacupuncture” in 2014, which is a top-cited animal experiment with 317 citations (Torres-Rosas et al., 2014). Using a mouse model of sepsis, they observed that sciatic nerve activation stimulated by low-intensity EA at ST36 controlled systemic inflammation and rescued the septic mice. Vagotomy or neurectomy of the sciatic nerve prevented the anti-inflammatory effects produced by EA, while vagus nerve stimulation mimicked the effect. Signals arising in the sciatic nerve activated the efferent vagus nerve, which induced dopamine release in the adrenal gland that targeted dopaminergic type 1 receptors and suppressed the production of inflammatory cytokines during sepsis. These results suggested a novel sciatic nerve-vagus nerve-adrenal pathway that regulated the innate immune response by dopamine production. The neuroimmune mechanism was not wholly sympathetic or parasympathetic (Chavan and Tracey, 2014). Liu CZ et al. found that EA performed at hindlimb regions inhibited the expression of GABA_A_ receptors in DMV neurons. Furthermore, excitation of the vagus nerve suppressed inflammation via activation of the α7nAChR-mediated JAK2/STAT3 signaling pathway in intestinal manipulation-induced postoperative ileus mice (Yang et al., 2021).

Ma Q and colleagues published two recent articles, “Somatotopic Organization and Intensity Dependence in Driving Distinct NPY-Expressing Sympathetic Pathways by Electroacupuncture” and “A neuroanatomical basis for electroacupuncture to drive the vagaladrenal axis” in *Neuron* and *Nature*, respectively, representing a milestone in acupuncture basic research (Liu et al., 2020, 2021). They demonstrated that EA drives two autonomic pathways and modulates lipopolysaccharide (LPS)-induced inflammation in somatotopy-, intensity-, and disease-state-dependent manners (Liu et al., 2020). High-intensity EA (3mA) at abdominal ST25 or hindlimb ST36 stimulates spinal sympathetic reflexes to activate the splenic sympathetic pathway producing anti- or pro-inflammatory effects based on “disease-state-dependent” changes. High-intensity EA stimulation before LPS injection evoked splenic noradrenergic neurons to prevent systemic inflammation via activation of the splenic cell β2 adrenergic receptors. In contrast, high-intensity EA performed after LPS injection when the cytokine storms have peaked produces a pro-inflammatory response via a2 adrenergic receptor signaling. Low-intensity EA (0.5mA) at the hindlimb ST36 can induce the vagal–adrenal axis to suppress ongoing systemic inflammation (Liu et al., 2020).

Ma Q and colleagues further explored the neuroanatomical basis for performing EA at specific acupoints (somatotopic organization) to stimulate the vagal-adrenal anti-inflammatory axis (Liu et al., 2021). PROKR2*^Cre^*-marked sensory neurons are abundant in the deep hindlimb fascia but not the abdominal fascia. Low-intensity EA at ST36 stimulated PROKR2*^Cre^*-marked sensory neurons to activate vagal efferent neurons and stimulate catecholamine release. Peritoneal fascia is critical for activating the vagal–adrenal axis. Low-intensity EA at ST36 in mice with ablated PROKR2*^Cre^*-marked sensory neurons could not activate vagal efferent neurons and increase the survival rate. Similarly, activation of PROKR2*^Cre^*-marked neurons mimics the anti-inflammatory effects of low-intensity EA at ST36. Stimulating the *Shousanli* (LI10) acupoint with the enrichment of PROKR2*^Cre^*-marked neurons could also evoke catecholamine production to reduce uncontrolled inflammation. This discovery allows either effective or ineffective anti-inflammatory effects of low-intensity EA in different body regions based on the distribution pattern of PROKR2*^Cre^*-marked nerves (Liu et al., 2021).

### Promising translational directions

Bioelectronic medicine is an emerging field in which selective modulation of ANS with electrons is applied to influence the function of well-characterized homeostatic reflexes as a replacement or supplement to conventional drug therapies (Pavlov et al., 2020; Cracchiolo et al., 2021; Pavlov and Tracey, 2022). Auricular acupuncture has been used for thousands of years in China. Afferent projections from the auricular branch of the vagus nerve to the NTS form the neuroanatomical basis for the vagal regulation by auricular acupuncture (He et al., 2012). Transcutaneous auricular vagus nerve stimulation (taVNS) is a promising non-invasive or minimally-invasive therapy in which the auricular branch of the vagus nerve is stimulated at the outer ear (Pavlov and Tracey, 2022). taVNS, a mechanism similar to auricular acupuncture, has become an established therapeutic option in the past two decades for treating various diseases (Wang et al., 2021). TEA also is a non-invasive method that replaces needles with surface electrodes and has been shown to improve GI motility (Zhang et al., 2021; Huang et al., 2022). Additional studies are needed to explore the mechanisms of taVNS and TEA and provide more evidence for clinical applications.

### Strengths and limitations

This is the first bibliometric analysis of the top 100 cited articles on the effects of acupuncture on autonomic function, which provides a comprehensive list of landmark publications and proof of the body of knowledge. The bibliometrix package in R was used to conduct analyses of publication patterns, country/region, institution, and author involvement. A well-known scientometric software tool (VOSviewer) was also used to visualize the bibliometric networks through co-citation analysis of journals and co-occurrence analysis of keywords. Nevertheless, some limitations should be noted. First, the 100 top-cited studies were obtained from the WoS database, which might exclude some top-cited studies from other databases. However, WoS does include complete information on citation records and is the most commonly used database in scientometrics. Second, recently published meaningful studies with low citations were not included in the final analysis (Yang et al., 2021; Zhang et al., 2021; Huang et al., 2022). However, after discovering this outcome, we read related research and discussed their results. And in future studies, we will fully utilize the citation per year for articles to obtain recently published impactful articles. Third, co-occurrence analysis results of keywords might be affected by the incomplete keyword extraction process of VOSviewer.

## Conclusion

Overall, this study of the 100 top-cited articles related to acupuncture’s regulation of autonomic function provides a comprehensive insight into landmark publications. The initial studies focused on the autonomic mechanism of visceral responses to acupuncture. Subsequently, researchers began to explore the autonomic mechanism of acupuncture control of systemic inflammation. Non-invasive electrical methods that stimulate somato-autonomic reflexes are new and critical directions in translational clinical medicine. However, additional studies on the underlying neuroanatomical basis of somato-autonomic reflexes are needed.

## Data availability statement

The original contributions presented in this study are included in the article/Supplementary material, further inquiries can be directed to the corresponding author.

## Author contributions

JG and ZZ contributed to conceptualization and writing the original draft. ZZ and RZ contributed to data collection and verification. LL, YY, CX, and RZ contributed to data analysis. LL and YY contributed to writing, review, and editing. All authors contributed to the article and approved the submitted version.
