# Supplementary material for: A bibliometric analysis of 100 top-cited journal articles related to acupuncture regulation of the autonomic nervous system
Source: Front Neurosci. 2022 Dec 22;16:1086087. doi: 10.3389/fnins.2022.1086087 (PMC9813952; doi:10.3389/fnins.2022.1086087)
Supplement: Supplementary file 1 [file Table_1.DOCX]

Supplementary Material

**Supplementary Table 1. The top 100 most-cited publications in acupuncture regulation of autonomic nervous system**

| **Rank** | **Article** | **TC** |
| --- | --- | --- |
| 1 | Zhang, RX, et al. Mechanisms of Acupuncture-Electroacupuncture on Persistent Pain. Anesthesiology.2014;120:482-503. | 345 |
| 2 | Andersson S, et al. Acupuncture--from empiricism to science: functional background to acupuncture effects in pain and disease. Med Hypotheses. 1995;45:271-81. | 323 |
| 3  4 | Torres-Rosas R, et al. Dopamine mediates vagal modulation of the immune system by electroacupuncture. Nat Med. 2014;20:291-5.  Dhond RP, et al. Acupuncture modulates resting state connectivity in default and sensorimotor brain networks. Pain. 2008;136:407-418. | 317  209 |
| 5 | Lin JG, et al. Acupuncture analgesia: a review of its mechanisms of actions. Am J Chin Med.2008;36:635-45. | 204 |
| 6  7  8  9 | Haker E, et al. Effect of sensory stimulation (acupuncture) on sympathetic and parasympathetic activities in healthy subjects. J Auton Nerv Syst. 2000;79:52-9.  Kotani N, et al. Preoperative intradermal acupuncture reduces postoperative pain, nausea and vomiting, analgesic requirement, and sympathoadrenal responses. Anesthesiology. 2001;95:349-56.  Sato A, et al. Neural mechanisms of the reflex inhibition and excitation of gastric motility elicited by acupuncture-like stimulation in anesthetized rats. Neurosci Res. 1993;18:53-62.  Kavoussi B, et al. The neuroimmune basis of anti-inflammatory acupuncture. Integr Cancer Ther.2007;6:251-7. | 195  173  164  152 |
| 10  11 | Takahashi T. Acupuncture for functional gastrointestinal disorders. J Gastroenterol.2006;41:408-17.  Nishijo K, et al. Decreased heart rate by acupuncture stimulation in humans via facilitation of cardiac vagal activity and suppression of cardiac sympathetic nerve. Neurosci Lett. 1997;227:165-8. | 151  136 |
| 12  13  14 | Ouyang H, et al. Electroacupuncture accelerates gastric emptying in association with changes in vagal activity. Am J Physiol Gastrointest Liver Physiol.2002;282:G390-6.  Stener-Victorin E, et al. Reduction of blood flow impedance in the uterine arteries of infertile women with electro-acupuncture. Hum Reprod. 1996;11:1314-7.  Knardahl S,et al.Sympathetic nerve activity after acupuncture in humans. Pain. 1998;75:19-25. | 134  124  118 |
| 15  16 | Stener-Victorin E, et al. Low-frequency electroacupuncture and physical exercise decrease high muscle sympathetic nerve activity in polycystic ovary syndrome. Am J Physiol Regul Integr Comp Physiol.2009;297:R387-95.  Chao DM, et al.Naloxone reverses inhibitory effect of electroacupuncture on sympathetic cardiovascular reflex responses. Am J Physiol. 1999;276:H2127-34. | 112  110 |
| 17 | Tatewaki M, et al. Dual effects of acupuncture on gastric motility in conscious rats. Am J Physiol Regul Integr Comp Physiol.2003;285:R862-72. | 98 |
| 18  19 | Chang FC, et al. The central serotonergic system mediates the analgesic effect of electroacupuncture on ZUSANLI (ST36) acupoints. J Biomed Sci. 2004 ;11:179-85.  Takeshige C, et al. Descending pain inhibitory system involved in acupuncture analgesia. Brain Res Bull. 1992;29:617-34. | 96  95 |
| 20 | Li Z, et al. Effects of acupuncture on heart rate variability in normal subjects under fatigue and non-fatigue state. Eur J Appl Physiol. 2005;94:633-40. | 92 |
| 21 | He W, et al. Auricular acupuncture and vagal regulation. Evid Based Complement Alternat Med. 2012;2012:786839. | 91 |
| 22 | Cho ZH, et al. Neural substrates, experimental evidences and functional hypothesis of acupuncture mechanisms. Acta Neurol Scand. 2006;113:370-7. | 90 |
| 23 | Tjen-A-Looi SC, et al. Medullary substrate and differential cardiovascular responses during stimulation of specific acupoints. Am J Physiol Regul Integr Comp Physiol. 2004;287:R852-62. | 90 |
| 24 | Li P, et al. Rostral ventrolateral medullary opioid receptor subtypes in the inhibitory effect of electroacupuncture on reflex autonomic response in cats. Auton Neurosci. 2001;89:38-47. | 89 |
| 25  26 | Sakai S, et al. Specific acupuncture sensation correlates with EEGs and autonomic changes in human subjects. Auton Neurosci. 2007;133:158-69.  Stener-Victorin E, et al. Effects of electro-acupuncture on nerve growth factor and ovarian morphology in rats with experimentally induced polycystic ovaries. Biol Reprod. 2000;63:1497-503. | 87  86 |
| 27  28  29 | Takahashi T. Mechanism of acupuncture on neuromodulation in the gut--a review. Neuromodulation. 2011;14:8-12.  Oke SL, et al. The inflammatory reflex and the role of complementary and alternative medical therapies. Ann N Y Acad Sci. 2009;1172:172-80.  Iwa M, et al. Electroacupuncture at ST-36 accelerates colonic motility and transit in freely moving conscious rats. Am J Physiol Gastrointest Liver Physiol. 2006;290:G285-92. | 85  85  83 |
| 30 | Kim SK, et al. Acupuncture and immune modulation. Auton Neurosci. 2010;157:38-41. | 80 |
| 31 | Huang ST, et al. Increase in the vagal modulation by acupuncture at neiguan point in the healthy subjects. Am J Chin Med. 2005;33:157-64. | 80 |
| 32 | Yin J, et al. Ameliorating effects and mechanisms of electroacupuncture on gastric dysrhythmia, delayed emptying, and impaired accommodation in diabetic rats. Am J Physiol Gastrointest Liver Physiol. 2010;298:G563-70. | 77 |
| 33 | Wang JD, et al. An alternative method to enhance vagal activities and suppress sympathetic activities in humans. Auton Neurosci. 2002;100:90-5. | 77 |
| 34  35  36 | Sato A, et al. Mechanism of the reflex inhibition of micturition contractions of the urinary bladder elicited by acupuncture-like stimulation in anesthetized rats. Neurosci Res. 1992;15:189-98.  Wang Q, et al. Electroacupuncture pretreatment attenuates cerebral ischemic injury through α7 nicotinic acetylcholine receptor-mediated inhibition of high-mobility group box 1 release in rats. J Neuroinflammation. 2012;9:24.  Streitberger K,et al. Acupuncture for nausea and vomiting: an update of clinical and experimental studies. Auton Neurosci. 2006;129:107-17. | 77  76  76 |
| 37 | Liu S, et al. Transcutaneous electroacupuncture improves dyspeptic symptoms and increases high frequency heart rate variability in patients with functional dyspepsia. Neurogastroenterol Motil. 2008;20:1204-11. | 75 |
| 38  39  40 | Lim HD, et al. Anti-Inflammatory Effects of Acupuncture Stimulation via the Vagus Nerve. PLoS One. 2016;11:e0151882.  Ohsawa H, et al.Neural mechanism of depressor responses of arterial pressure elicited by acupuncture-like stimulation to a hindlimb in anesthetized rats. J Auton Nerv Syst. 1995;51:27-35.  Chen J, et al. Electroacupuncture improves impaired gastric motility and slow waves induced by rectal distension in dogs. Am J Physiol Gastrointest Liver Physiol. 2008;295:G614-20. | 73  73  70 |
| 41  42 | Sandberg M, et al. Different patterns of blood flow response in the trapezius muscle following needle stimulation (acupuncture) between healthy subjects and patients with fibromyalgia and work-related trapezius myalgia. Eur J Pain. 2005;9:497-510.  Tjen-A-Looi SC, et al. Prolonged inhibition of rostral ventral lateral medullary premotor sympathetic neurons by electroacupuncture in cats. Auton Neurosci. 2003;106:119-31. | 70  70 |
| 43  44  45 | Shen J. Research on the neurophysiological mechanisms of acupuncture: review of selected studies and methodological issues. J Altern Complement Med. 2001;7 :S121-7.  Noguchi E, et al. The effect of electro-acupuncture stimulation on the muscle blood flow of the hindlimb in anesthetized rats. J Auton Nerv Syst. 1999;75:78-86.  Tjen-A-Looi SC, et al. Midbrain vlPAG inhibits rVLM cardiovascular sympathoexcitatory responses during electroacupuncture. Am J Physiol Heart Circ Physiol. 2006;290:H2543-53. | 69  69  68 |
| 46 | Mannerås L, et al. Acupuncture and exercise restore adipose tissue expression of sympathetic markers and improve ovarian morphology in rats with dihydrotestosterone-induced PCOS. Am J Physiol Regul Integr Comp Physiol. 2009;296:R1124-31. | 65 |
| 47 | Zhao YX, et al. Transcutaneous auricular vagus nerve stimulation protects endotoxemic rat from lipopolysaccharide-induced inflammation. Evid Based Complement Alternat Med. 2012;2012:627023. | 64 |
| 48 | Stener-Victorin E,et al. Acupuncture in polycystic ovary syndrome: current experimental and clinical evidence. J Neuroendocrinol. 2008;20:290-8. | 64 |
| 49 | Ulloa L, et al. Nerve Stimulation: Immunomodulation and Control of Inflammation. Trends Mol Med. 2017;23:1103-1120. | 63 |
| 50 | Jin H, et al. Anti-inflammatory effects and mechanisms of vagal nerve stimulation combined with electroacupuncture in a rodent model of TNBS-induced colitis. Am J Physiol Gastrointest Liver Physiol. 2017;313:G192-G202. | 63 |
| 51 | Middlekauff HR, et al. Acupuncture inhibits sympathetic activation during mental stress in advanced heart failure patients. J Card Fail. 2002;8:399-406. | 63 |
| 52 | Mori H, et al. Unique immunomodulation by electro-acupuncture in humans possibly via stimulation of the autonomic nervous system. Neurosci Lett. 2002;320:21-4. | 62 |
| 53 | Imai K, et al. Effects of electroacupuncture on gastric motility and heart rate variability in conscious rats. Auton Neurosci. 2008 ;138:91-8. | 61 |
| 54 | Li YQ, et al. Neural mechanism of acupuncture-modulated gastric motility. World J Gastroenterol. 2007;13:709-16. | 61 |
| 55  56 | Liu S, et al. Somatotopic Organization and Intensity Dependence in Driving Distinct NPY-Expressing Sympathetic Pathways by Electroacupuncture. Neuron. 2020;108:436-450.  Lux G,et al. Acupuncture inhibits vagal gastric acid secretion stimulated by sham feeding in healthy subjects. Gut. 1994;35:1026-9. | 60  60 |
| 57  58  59 | Lee B, et al. Acupuncture stimulation improves scopolamine-induced cognitive impairment via activation of cholinergic system and regulation of BDNF and CREB expressions in rats. BMC Complement Altern Med. 2014;14:338.  Gao XY, et al. Investigation of specificity of auricular acupuncture points in regulation of autonomic function in anesthetized rats. Auton Neurosci. 2008;138:50-6.  Bäcker M, et al. Acupuncture in migraine: investigation of autonomic effects. Clin J Pain. 2008;24:106-15. | 59  59  58 |
| 60 | Stener-Victorin E, et al. Ovarian blood flow responses to electroacupuncture stimulation depend on estrous cycle and on site and frequency of stimulation in anesthetized rats. J Appl Physiol (1985). 2006;101:84-91. | 58 |
| 61 | Liu S, et al. A neuroanatomical basis for electroacupuncture to drive the vagal-adrenal axis. Nature. 2021;598:641-645. | 57 |
| 62 | Manni L, et al. Effect of electro-acupuncture on ovarian expression of alpha (1)- and beta (2)-adrenoceptors, and p75 neurotrophin receptors in rats with steroid-induced polycystic ovaries. Reprod Biol Endocrinol. 2005;3:21. | 57 |
| 63 | Tada H, et al. Neural mechanism of acupuncture-induced gastric relaxations in rats. Dig Dis Sci. 2003;48:59-68. | 57 |
| 64  65 | Zhou WY, et al. Brain stem mechanisms underlying acupuncture modality-related modulation of cardiovascular responses in rats. J Appl Physiol (1985). 2005;99:851-60.  Shiraishi T, et al. Effects of auricular stimulation on feeding-related hypothalamic neuronal activity in normal and obese rats. Brain Res Bull. 1995;36:141-8. | 56  56 |
| 66  67 | Li P, et al. Neural mechanism of electroacupuncture's hypotensive effects. Auton Neurosci. 2010;157:24-30.  Stener-Victorin E, et al. Ovarian blood flow responses to electro-acupuncture stimulation at different frequencies and intensities in anaesthetized rats. Auton Neurosci. 2003;108:50-6. | 55  55 |
| 68 | Guo ZL, et al. Electroacupuncture induces c-Fos expression in the rostral ventrolateral medulla and periaqueductal gray in cats: relation to opioid containing neurons. Brain Res. 2004;1030:103-15. | 54 |
| 69 | Zhou W, et al. Neuroendocrine mechanisms of acupuncture in the treatment of hypertension. Evid Based Complement Alternat Med. 2012;2012:878673. | 53 |
| 70 | Loaiza LA, et al. Electro-acupuncture stimulation to muscle afferents in anesthetized rats modulates the blood flow to the knee joint through autonomic reflexes and nitric oxide. Auton Neurosci. 2002;97:103-9. | 53 |
| 71 | Zhang Y, et al. Electroacupuncture inhibition of hyperalgesia in an inflammatory pain rat model: involvement of distinct spinal serotonin and norepinephrine receptor subtypes. Br J Anaesth. 2012;109:245-52. | 52 |
| 72 | Lee S, et al. Acupuncture and heart rate variability: a systematic review. Auton Neurosci. 2010;155:5-13. | 52 |
| 73  74 | Sato A, et al. Reflex modulation of catecholamine secretion and adrenal sympathetic nerve activity by acupuncture-like stimulation in anesthetized rat. Jpn J Physiol. 1996;46:411-21.  Kim JH, et al. Electroacupuncture acutely improves cerebral blood flow and attenuates moderate ischemic injury via an endothelial mechanism in mice. PLoS One. 2013;8:e56736. | 52  51 |
| 75 | Gao XY, et al. Acupuncture-like stimulation at auricular point Heart evokes cardiovascular inhibition via activating the cardiac-related neurons in the nucleus tractus solitarius. Brain Res. 2011;1397:19-27. | 50 |
| 76  77 | Tjen-A-Looi SC,et al. Role of medullary GABA, opioids, and nociceptin in prolonged inhibition of cardiovascular sympathoexcitatory reflexes during electroacupuncture in cats. Am J Physiol Heart Circ Physiol. 2007;293:H3627-35.  Sugiyama Y, et al. Transient increase in human muscle sympathetic nerve activity during manual acupuncture. Jpn J Physiol. 1995;45:337-45. | 50  50 |
| 78 | Song JG, et al. Electroacupuncture improves survival in rats with lethal endotoxemia via the autonomic nervous system. Anesthesiology. 2012;116:406-14. | 49 |
| 79 | Luo D, et al. Electroacupuncture at acupoint ST-36 promotes contractility of distal colon via a cholinergic pathway in conscious rats. Dig Dis Sci. 2008;53:689-93. | 49 |
| 80 | Liu JH, et al.Effects of electroacupuncture on gastric myoelectric activity and substance P in the dorsal vagal complex of rats. Neurosci Lett. 2004;356:99-102. | 49 |
| 81 | Lee SY, et al. Intradermal acupuncture on shen-men and nei-kuan acupoints improves insomnia in stroke patients by reducing the sympathetic nervous activity: a randomized clinical trial. Am J Chin Med. 2009;37:1013-21. | 48 |
| 82 | Kim HW, et al. Low-frequency electroacupuncture suppresses carrageenan-induced paw inflammation in mice via sympathetic post-ganglionic neurons, while high-frequency EA suppression is mediated by the sympathoadrenal medullary axis. Brain Res Bull. 2008;75:698-705. | 46 |
| 83 | Napadow V, et al. Brain correlates of phasic autonomic response to acupuncture stimulation: an event-related fMRI study. Hum Brain Mapp. 2013;34:2592-606. | 44 |
| 84 | Streitberger K, et al. Effects of verum acupuncture compared to placebo acupuncture on quantitative EEG and heart rate variability in healthy volunteers. J Altern Complement Med. 2008;14:505-13. | 44 |
| 85 | Schneider A, et al.Neuroendocrinological effects of acupuncture treatment in patients with irritable bowel syndrome. Complement Ther Med. 2007;15:255-63. | 44 |
| 86 | Sallam H, et al. Transcutaneous electrical nerve stimulation (TENS) improves upper GI symptoms and balances the sympathovagal activity in scleroderma patients. Dig Dis Sci. 2007;52:1329-37. | 44 |
| 87  88 | Bucinskaite V, et al. Effects of repeated sensory stimulation (electro-acupuncture) and physical exercise (running) on open-field behaviour and concentrations of neuropeptides in the hippocampus in WKY and SHR rats. Eur J Neurosci. 1996;8:382-7.  Eshkevari L, et al. Acupuncture blocks cold stress-induced increases in the hypothalamus-pituitary-adrenal axis in the rat. J Endocrinol. 2013;217:95-104. | 43  42 |
| 89 | Huang W, et al. Autonomic activation in insomnia: the case for acupuncture. J Clin Sleep Med. 2011;7:95-102. | 42 |
| 90 | Noguchi E. Acupuncture regulates gut motility and secretion via nerve reflexes. Auton Neurosci. 2010;156:15-8. | 42 |
| 91 | Moazzami A, et al. Serotonergic projection from nucleus raphe pallidus to rostral ventrolateral medulla modulates cardiovascular reflex responses during acupuncture. J Appl Physiol (1985). 2010;108:1336-46. | 41 |
| 92 | Middlekauff HR, et al. Acupuncture effects on reflex responses to mental stress in humans. Am J Physiol Regul Integr Comp Physiol. 2001;280:R1462-8. | 41 |
| 93 | Hu S, et al. Electroacupuncture at Zusanli (ST36) Prevents Intestinal Barrier and Remote Organ Dysfunction following Gut Ischemia through Activating the Cholinergic Anti-Inflammatory-Dependent Mechanism. Evid Based Complement Alternat Med. 2013;2013:592127. | 40 |
| 94 | Iwa M, et al.Anatomical evidence of regional specific effects of acupuncture on gastric motor function in rats. Auton Neurosci. 2007;137:67-76. | 40 |
| 95 | Iwa M, et al. Electroacupuncture elicits dual effects: stimulation of delayed gastric emptying and inhibition of accelerated colonic transit induced by restraint stress in rats. Dig Dis Sci. 2006;51:1493-500. | 40 |
| 96 | Tsou MT, et al. Electroacupuncture on PC6 (Neiguan) attenuates ischemia/reperfusion injury in rat hearts. Am J Chin Med. 2004;32:951-65. | 40 |
| 97 | Beissner F, et al. Acupuncture--deep pain with an autonomic dimension? Neuroimage. 2012;60:653-60. | 39 |
| 98 | Du MH, et al.Electroacupuncture improves gut barrier dysfunction in prolonged hemorrhagic shock rats through vagus anti-inflammatory mechanism. World J Gastroenterol. 2013;19:5988-99. | 38 |
| 99 | Liu RP, et al.Effects of electroacupuncture at auricular concha region on the depressive status of unpredictable chronic mild stress rat models. Evid Based Complement Alternat Med. 2013;2013:789674. | 37 |
| 100 | Cheng KJ. Neuroanatomical basis of acupuncture treatment for some common illnesses. Acupunct Med. 2009;27:61-4. | 37 |

TC: total citation

**Supplementary Figure 1.** The flowchart of publications selection.

**
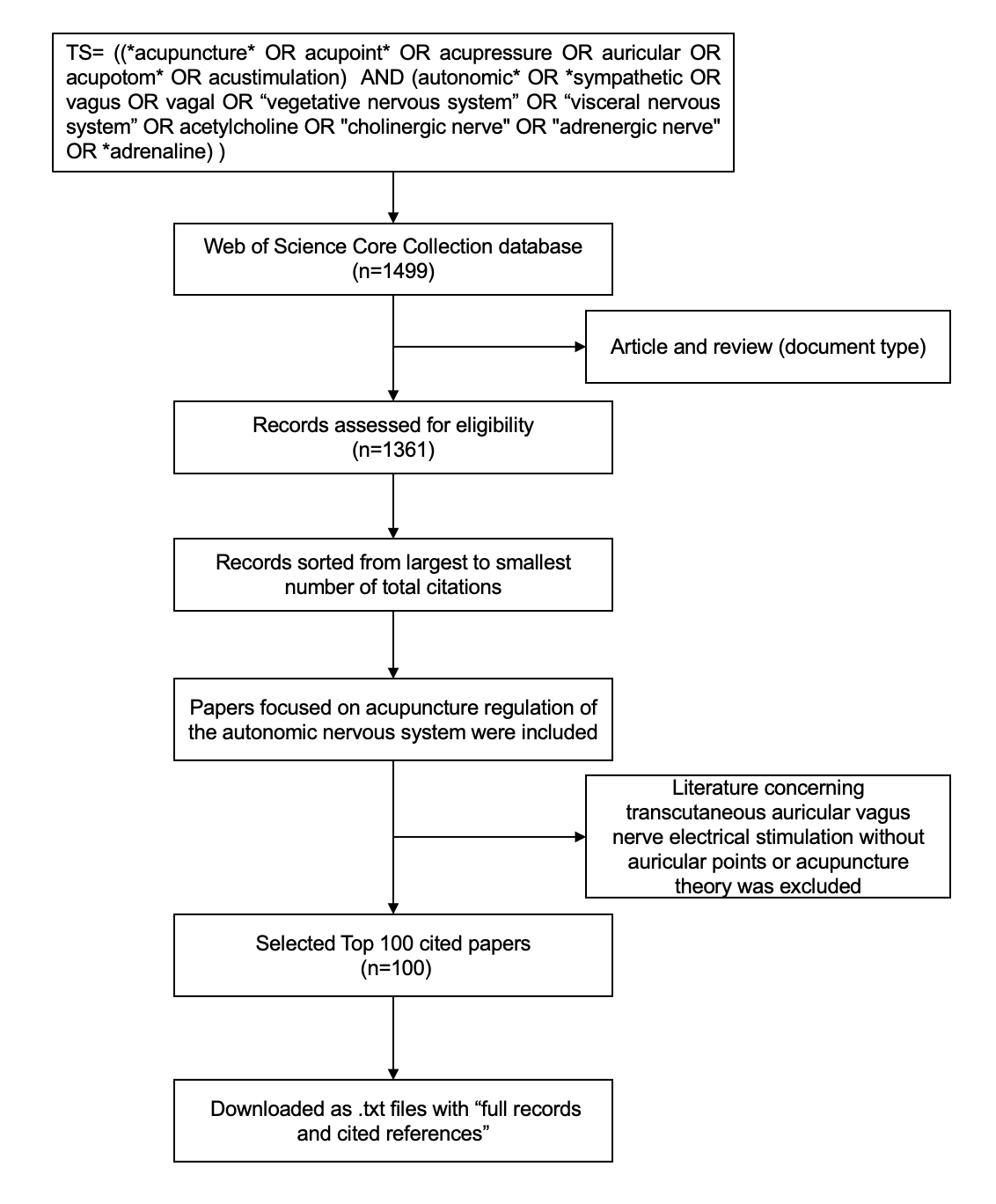
**
